# Preliminary Evaluation of an Indigenous Mental Health App for Indian Users

**DOI:** 10.7759/cureus.80346

**Published:** 2025-03-10

**Authors:** Abhishek Karishiddimath, Angelina Francis, Hridya Harikumar, Pramita Sengupta, T.K. Srikanth, Seema Mehrotra

**Affiliations:** 1 Clinical Psychology, National Institute of Mental Health and Neuro Sciences, Bengaluru, IND; 2 E-Health Research Centre, International Institute of Information Technology Bangalore, Bengaluru, IND

**Keywords:** digital mental health, india, indian app, mental health app, mhapp

## Abstract

Background

The digital health sector has witnessed significant growth in recent years. There are many mental health apps available in virtual stores for Indian users; however, there is a dearth of research on the same. The present study aimed at a preliminary examination of the usability, acceptability, and perceived gains of an indigenously developed app (MindNotes, National Institute of Mental Health and Neuro Sciences, Bengaluru, India) for common mental health concerns.

Method

A short-term prospective design was employed. A sample of 190 community participants completed a baseline survey and were suggested to explore the app for two weeks. The baseline survey examined self-reports of current common mental health concerns, their duration and perceived severity, and their commitment to try the app during the study period. The inclination to seek help from a mental health professional was inquired into using a seven-point Likert scale. A post-assessment survey was conducted in the third week to capture the extent of app exploration, frequency of usage, and ratings of the participants on perceived usefulness, engaging nature, ease of navigation, likelihood of future use, and recommending the app to others. The item on inclination to seek help from a mental health professional was repeated. Perceived gains were examined through responses to a checklist. In addition to descriptive statistics such as frequencies and percentages for identifying patterns of responses to various items, correlational analysis was used to examine relationships between ease of navigation and the engaging nature of the app with the extent of exploration and the likelihood of future use of the app. The Mann-Whitney U test was used to examine the difference in the extent of app exploration between subgroups with varying levels of baseline commitment to use the app, while the Wilcoxon signed-rank test was used to examine changes in help-seeking inclination from baseline to post-assessment. Predictors of help-seeking inclination at post-assessment were examined through a hierarchical regression analysis.

Result

Based on the responses to the survey items, the app was found to be easy to navigate and engaging by 83 (76.2%) and 84 (77.1%) participants, respectively, and received moderate to high ratings from 95 participants (87.2%) on perceived usefulness. The extent of app exploration significantly predicted improvement in help-seeking inclination at post-assessment in a subgroup of participants reporting common mental health concerns.

Conclusion

The findings of this preliminary study support the usability, potential usefulness, and acceptability of MindNotes, a multi-module mental health app for common mental health concerns developed for Indian users. There is a high treatment gap for common mental health problems in India, which is partly attributable to various barriers to seeking professional help, including stigma. The study results suggest that the MindNotes app may have utility in improving the inclination to seek professional help in individuals experiencing common mental health concerns and thereby may serve as one of the approaches to address the treatment gap.

## Introduction

In the background of the high prevalence of common mental health conditions and the huge treatment gap noted worldwide, the World Health Organization (WHO) proposed an optimum mix for organizing services in the form of a pyramid. It highlights that most individuals in any given population suffer from milder severity conditions that can be effectively managed with lower-intensity interventions such as self-help interventions and peer- and community-based support as compared to highly specialized services that are needed by a lower proportion of individuals in the population [1].

In India, the treatment gap for common mental disorders has been estimated to be 85% [2]. A variety of factors contribute to this treatment gap such as a lack of easy access to appropriate mental health care and insufficient or inequitably distributed resources (supply-side barriers) as well as demand-side barriers (low rates of help-seeking) such as low mental health literacy, stigma, low perceived need, and attitudinal factors [2-4].

With the growing recognition of the role of technology in meeting the unmet mental health needs of the population, the last few decades have witnessed rapid advancements in the use of internet-based programs and self-help apps to address the unmet mental health needs in multiple ways such as an adjunct to mainstream services, while waiting to receive mainstream care, for symptom monitoring, relapse prevention, and improvement of mental health awareness, as well as for use of a standalone self-help tool for milder problems [5,6]. Technology-based interventions have also been examined for their utility in improving help-seeking for mental health [7].

Voluminous research has been generated on unguided or purely self-driven digital interventions as well as guided digital interventions (involving varying elements of human support) that target a variety of mental health conditions/concerns, and now, there is a plethora of systematic reviews and meta-analyses, particularly from developed nations [8,9]. Despite this, there is a preponderance of mental health apps in commercial marketplaces that have not been researched or on which there is little systematic examination of the use of evidence-based content [10-12]. This phenomenon is even more pronounced in the rapidly evolving Indian digital mental health field [13].

It is in this background that a mobile app was developed for Indian users, with the aim of empowering individuals with common mental health concerns with interactive, evidence-based content to enhance clarity about the nature and severity of mental health concerns and reduce ambivalence about seeking professional help when required, while also learning to use self-help strategies through modules of relevance to persons experiencing mood and anxiety symptoms or psychological distress in general. The app content and features were designed based on an extensive review of literature and clinical and research experience of the mental health professionals who conceptualized the app as well as formative research by the team [14]. It has multiple sections. The Self-discovery section contains quizzes and story-based questions to help users gain self-awareness about their mental health concerns and receive feedback. The Self-help section has seven modules containing simple exercises and strategies on themes such as reducing self-criticality, mastering worry, behavioral activation, calming and soothing, managing negative thoughts, managing anger, and managing social anxiety. The Breaking barriers section helps users to identify the mental blocks/hesitations they may have about seeking professional help for mental health and offers brief inputs to overcome such barriers. The Coping with crisis section includes simple strategies for dealing with emotional crises by developing a crisis response plan and accessing helpline numbers. The Professional connect section allows users to send brief notes to a panel of mental health professionals to clarify general doubts about therapy or other forms of treatment. This app is freely available on Google Play Store (Google, Mountain View, CA, US) as a noncommercial offering by the National Institute of Mental Health and Neuro Sciences (NIMHANS), India, and hence called MindNotes from NIMHANS app.

Rationale

There is a severe scarcity of research on indigenously developed mental health apps in India. Although there has been tremendous growth in the digital mental health market in India with many startups in the field, concerns have been raised about issues such as quality of content, their validation by domain experts (mental health professionals), and privacy and security apart from insufficient documentation on usability and effectiveness for the professed purposes. In this background, the present study was designed to address some of these gaps in the Indian digital mental health space by carrying out a pilot evaluation of the indigenously developed app.

Objectives

The present study aimed at a preliminary evaluation of the self-reported usage, usability, perceived usefulness, and acceptability of the MindNotes app, presented as a self-help digital tool for mental health. In addition, it examined changes in inclination to seek professional help following two weeks of app exploration in a subgroup of participants with self-reports of common mental health concerns and evaluated the role of the extent of exploration of the app as a predictor of this change.

## Materials and methods

This preliminary study is part of an ongoing work focused on developing a technology-based platform for promoting self-care and help-seeking in individuals with common mental health concerns in India. Ethical approval was duly obtained from the institutional ethics committee (NIMHANS/EC (Beh.Sc.Div.) 17th Meeting/2019). Informed consent was collected from all participants. A short-term prospective design was used to address the objectives.

Procedure

The study utilized a convenience sampling approach for this preliminary work. It was announced via recruitment flyers on various online social media platforms such as Facebook and Instagram as well as during mental health outreach programs between February and April 2024 in various educational campuses in five districts of the state of Karnataka in India as part of ongoing projects. The announcements solicited participation from those who were interested in learning about “Self-help” to improve mental well-being. Individuals 18 years or older, comfortable in reading and writing in English or Kannada, having access to an Android phone, and open to using a smartphone app to learn self-help strategies for improving mental well-being were invited to participate. Those interested were directed to provide informed consent and subsequently complete a baseline survey online. The survey did not contain the name of the app to minimize bias, and upon completion of the survey, the participants were directed to a QR code to download the app and explore the same for a period of two weeks. WhatsApp (Meta Platforms, Inc., Menlo Park, CA, US) reminders were sent to the participants weekly to encourage engagement with the app. In the third week, a post-assessment Google survey link was mailed and sent over WhatsApp to the participants. Two reminders were sent for completing the post-assessment survey during the third week.

Assessment surveys

A baseline survey was developed for the study, which included the following components in addition to the basic sociodemographic details such as age, gender, education, and occupational status. Past use of any internet-based program/app for health needs in general was documented through a dichotomous item. One item each on self-rated perseverance in pursuing self-set daily goals and interest in learning self-help skills for managing one’s mental health needs (five-point Likert scale) were used as these are relevant for studies examining engagement with mental health self-help apps [15]. Self-report of current common mental health concerns was elicited through an item involving a multiple-choice format: “Which of the following best describes your current mental health state? a) I have been frequently feeling nervous/tense/worried/anxious and not able to relax, b) I have been feeling sad most of the time/less interested in the things that I used to enjoy earlier, c) Both, and d) None.” The duration of current mental health concern was also inquired with a multiple-choice item: a) Less than two weeks, b) About two weeks, c) Two weeks to one month, d) One month to three months, e) Three months to six months, and f) More than six months. In addition, two five-point Likert scale items were used to capture a) the self-reported severity of current mental health concerns and b) the extent of helpfulness of self-help methods being used for the same. The inclination to seek help from a mental health professional for one's concerns was assessed using a seven-point Likert scale from “extremely likely” to “extremely unlikely.” This has been found to be sensitive to change following help-seeking intervention in prior research [16]. Lastly, commitment to trying out the app-based self-help over the two-week study period was inquired into (yes certainly/yes to some extent/unsure).

The post-survey comprised items to explore the following: five-point Likert-type item for a) extent of exploration of the app modules during the study period (hardly to almost fully) and b) frequency of app usage (hardly to more than five times). Barriers to using the app more often were solicited through a multiple-choice item (did not find it relevant/was just curious/wanted to but could not find time). The perceived usefulness of the explored modules, engaging nature, ease of navigation, and the likelihood of using the modules/applying them in the future were inquired into via five-point Likert-type items. In addition, two items asked about the perceived relevance of the app content and its usefulness for persons experiencing mental health concerns/distress but not seeking help using five-point Likert-type ratings (very poor to excellent). Two baseline survey items on the severity of current concerns and help-seeking inclination were repeated at post-assessment. Self-report of any change in motivation to seek professional help for mental health was captured using a multiple-choice item. Perceived gains from using the app were documented using a checklist developed based on gains that may be expected out of app use (i.e., improved awareness about mental health). The post-survey also inquired about the likelihood of recommending the app to family or friends who may be experiencing distress but not seeking help (yes/no/unsure). The participants were also requested to share any feedback, suggestions, or recommendations for the app through an open-ended item at the end of the post-assessment survey.

Statistical analyses

Frequencies and percentages were used to depict the pattern of responses on various items of the survey. Spearman's correlations were used to examine correlations among variables, i.e., ease of navigation, engaging nature, extent of exploration, and likelihood of future use of the app. The Mann-Whitney U test was used to examine differences in terms of the extent of exploration of the app between subgroups with different levels of baseline commitment to use the app. Furthermore, changes in inclination to seek help from professionals from baseline to post-assessment were examined using the Wilcoxon signed-rank test. Hierarchical regression analysis was performed to examine the predictors of help-seeking inclination at the post-assessment.

## Results

There were 190 participants who provided informed consent and completed the baseline survey. The details of this sample are as described below.

There was a preponderance of young adults and women participants in the sample as noted in Table 1. The majority of the sample consisted of students while working adults formed slightly less than one-fifth of the sample.

**Table 1:** Sociodemographic characteristics of the sample at baseline

Characteristics	n (%)
Age (years)	
18-35	163 (85.8%)
36-53	22 (11.6%)
54 above	5 (2.6%)
Gender	
Male	38 (20%)
Female	152 (80%)
Highest level of education completed	
Matriculation	6 (3.2%)
Pre-University/diploma	15 (7.9%)
Under graduation	119 (62.6%)
Post-graduation/above	50 (26.3%)
Occupational status	
Student	150 (78.9%)
Seeking a job	3 (1.6%)
Employed	26 (13.7%)
Self-employed	6 (3.2%)
Homemaker	3 (1.6%)
Retired	2 (1.1%)

Of the participants, 16%-25% endorsed the presence of depressive and anxiety symptoms, respectively, and another one-third reported experiencing a mix of depressive and anxiety symptoms. About one-third reported the duration of these symptoms to be between two weeks and six months, while 60 (31%) participants reported to be experiencing these symptoms for more than six months. While about 109 (57%) participants rated their severity of distress to be relatively low (1/2 on the five-point scale), 56 (30%) participants rated it at the moderate level (3), and the remaining 25 (13%) rated it to be high (4/5) (Table 2).

**Table 2:** Other sample characteristics at baseline ^*^The severity level has been depicted by adding responses on the five-point Likert scale. Responses involving the first two options and the last two items on the Likert scale were collapsed to depict low and high severity, respectively, whereas responses marked as “3” are mentioned as moderate.

Variables	n (%)
Current mental state	
Have been feeling sad most of the time/less interested in the things enjoyed earlier	30 (15.8)
Frequently feeling nervous/tensed/worried/anxious and unable to relax	48 (25.3)
Both of the above	62 (32.6)
None	50 (26.3)
Duration of current mental health concerns	
Less than two weeks	47 (24.7)
About two weeks	20 (10.5)
Two weeks to one month	31 (16.3)
One month to three months	13 (6.8)
Three months to six months	19 (10)
More than six months	60 (31.6)
Severity of current mental health concerns^*^	
Low	109 (57.4)
Moderate	56 (29.5)
Severe	25 (13.1)
Interested in learning self-help skills for mental health	
Very interested	129 (67.9)
Somewhat interested	46 (24.2)
Maybe/unsure	5 (2.6)
Not much interested	3 (1.6)
Hardly interested	7 (3.7)
Ever used internet-based programs/app for health	
Yes	61 (32.1)
No	129 (67.9)
Extent of the usefulness of self-help methods for current concerns	
Not at all helped	15 (7.9)
Helped slightly	56 (29.5)
Helped to some extent	80 (42.1)
Helped to a great extent	31 (16.3)
Helped extremely	8 (4.2)

The majority, 129 (67%), were very interested in learning self-help skills to manage mental health concerns. About two-thirds of the study participants had not used any internet-based programs/apps for health concerns in general. Furthermore, 71 (37%) participants indicated that self-help methods had not been helpful or had helped only slightly in managing current mental health concerns (Table 2).

In addition, 103 (54.2%) participants reported themselves to be high on perseverance in pursuing self-set daily goals while 87 (45.8%) reported being low on the same. In terms of commitment to try the app during the study period, 105 (55.2%) indicated that they were certainly committed, while 74 (39%) indicated that they were committed to some extent and 11 (5.8%) participants were unsure about commitment to using the app during the study period.

Out of 190 participants who initially enrolled in the study, 109 (57%) participants returned the completed post-assessment survey by the end of the third week. Further analyses were based on this sample of completers.

While slightly more than one-third reported having been able to explore the app to some extent, 31 (29%) participants had explored it to a large extent/almost fully. In terms of frequency of usage during the study period, 18 (17%) participants reported having used it more than thrice, and 52 (48%) participants used it two to three times. On the other hand, slightly more than a quarter indicated having used the app only once. Time constraints were cited as the commonest barrier to more frequent usage, followed by signing up merely out of curiosity. Irrelevant content was endorsed as a reason for infrequent use by only about five (5%) participants (Table 3).

**Table 3:** App usage during the study period and feedback ^*^The low, moderate, and high categories are mentioned by collapsing two extreme responses at each end to form low/high subgroups, respectively, while retaining “3” as it is to indicate moderate levels.

Variables	n (%)
Able to explore the chosen sections	
Hardly	8 (7.3%)
To a small extent	30 (27.5%)
To some extent	40 (36.7%)
To quite an extent	22 (20.2%)
Almost fully	9 (8.3%)
Frequency of the usage of the chosen sections	
Hardly	7 (6.4%)
Once	31 (28.4%)
Two to three times	52 (47.7%)
Four to five times	18 (16.5%)
More than five times	1 (0.9%)
Barriers to using the chosen sections	
Irrelevant	5 (4.6%)
Signed up just out of curiosity	37 (33.9%)
Did not have time	60 (55.0%)
Other reasons	7 (6.4%)
Likelihood of using the app in the future^*^	
Low	15 (13.8%)
Moderate	35 (32.1%)
High	59 (54.1%)
Perceived usefulness	
Low	14 (12.8%)
Moderate	45 (41.3%)
High	50 (45.9%)
Engaging nature	
Low	25 (22.9%)
Moderate	41 (37.6%)
High	43 (39.5%)
Ease of navigation	
Low	26 (23.8%)
Moderate	41 (37.6%)
High	42 (38.6%)

The app was rated as high on engaging nature by 43 (40%) participants; 42 (39%) participants rated it to be high on ease of navigation while high usefulness was endorsed by 50 (46%) participants. On the other hand, lower ratings on engaging nature and navigation ease were given by nearly a quarter of the participants. Fourteen (13%) participants rated the app to be low in usefulness (Table 3).

As per self-reports, a quarter reported being motivated to seek help for mental health after using the app, whereas 34 (30%) participants reported increased motivation to seek help though they were yet to engage in help-seeking at the end of the study. Moreover, 15 (14%) participants reported that the app did not impact their motivation to seek help, and 10 (10%) participants indicated that they did not need to seek such help (Table 4).

**Table 4:** Perceived impact of app usage and perceived utility for distressed nontreatment seekers

Variables	n (%)
Perceived motivational impact	
Felt motivated to seek help after using the app and took the help	27 (24.8%)
Took help but was planning to seek help anyway	15 (13.8%)
Felt motivated but have yet to find a suitable professional	16 (14.7%)
Felt motivated but going to seek help in the near future	18 (16.5%)
No, the app did not increase my motivation	15 (13.8%)
No, I did not have any need to seek professional help	10 (9.2%)
No, I did not use the app	8 (7.3%)
Perceived gains	
My exploration has increased my overall knowledge of mental well-being	14 (12.8%)
Feel more open and positive about utilizing professional help	13 (11.9%)
Feel that I learned some useful strategies from MindNotes	11 (10.1%)
Feel more confident about applying a few simple coping strategies	16 (14.7%)
MindNotes has helped in feeling less distressed	9 (8.3%)
I feel no change	5 (4.6%)
Did not use the app	10 (9.2%)
Combinations of the above options except involving any of these: “Did not use the app” and "I feel no change"	28 (25.7%)
Perceived relevance of content	
Low	7 (6.4%)
Moderate	24 (22.0%)
High	78 (71.6%)
Perceived usefulness of content	
Low	10 (9.2%)
Moderate	32 (29.4%)
High	67 (61.5%)
Likelihood of recommending MindNotes	
Yes	64 (58.7%)
Maybe	35 (32.1%)
No	10 (9.2%)

In addition, 28 (26%) participants endorsed more than one perceived gain from using the app in terms of increased knowledge, openness to utilizing professional help, learning strategies for self-help, increased efficacy in applying coping strategies, or feeling less distressed (Table 4).

Additionally, 67 (62%) participants rated the app highly on perceived usefulness, and 78 (72%) participants rated the app highly on perceived relevance of app content for persons in distress who may not be seeking professional help. Also, 64 (59%) participants reported that they were certain they would recommend the app to others in their circle who were going through emotional distress/mental health difficulties while 32% reported being unsure of the same (Table 4).

Ratings on the app's ease of navigation and engaging nature showed significant positive correlations with perceived usefulness (r = 0.38-0.62, p < 0.01) and the likelihood of future use (r = 0.45-0.52, p < 0.01). The ratings on the engaging nature of the app demonstrated a positive correlation with the extent of exploration of the app (r = 0.29, p < 0.01).

In addition, in a supplementary analysis, it was noted that participants reporting higher commitment to using the app at baseline reported significantly higher exploration of the app at post-assessment than those with lower commitment (z = 3.65, p < 0.001).

Changes in inclination to seek help from professionals from baseline to post-assessment were examined in the subgroup of individuals who self-reported depressive symptoms, anxiety symptoms, or a combination of the same at baseline. The mean help-seeking inclination significantly increased from 3.42 (SD = 1.96) at baseline to 4.62 (SD = 1.72) post-assessment, with a Wilcoxon signed-rank test indicating a significant increase in help-seeking inclination noted in this group at post-assessment (z = 4.47, p < 0.01).

Furthermore, a hierarchical linear regression analysis was carried out to examine the role of the extent of exploration of the app as a predictor of help-seeking inclination at post-assessment while controlling for help-seeking inclination at baseline, age, gender, and duration of self-reported symptoms at baseline as well as self-perceived severity of distress at post-assessment. It was noted that the extent of exploration of the app was a significant predictor of help-seeking inclination at post-assessment, over and above other variables including perceived severity of distress at post-assessment (Table 5).

**Table 5:** Predictors of help-seeking inclination at post-assessment: hierarchical regression ^*^p < 0.05, ^**^p < 0.01, ^***^p < 0.001; final model R² = 0.34; adjusted R² = 0.29; F = 6.15^**^

Predictors	Unstandardized coefficients		Standardized coefficients	
					B	Std. error	Beta		
Step 1					
Constant	3.69	0.90			
Age	-0.02	0.03	-0.08		
Gender	0.09	0.46	0.02		
Duration of concern	0.22	0.40	0.06		
Inclination to help-seeking (at baseline)	0.27	0.10	0.31^**^		
Step 2					
Constant	1.51	0.94			
Age	-0.02	0.02	-0.07		
Gender	0.23	0.41	0.56		
Duration	0.22	0.36	0.06		
Inclination to help-seeking (at baseline)	0.17	0.09	0.20		
Severity of concern (post-assessment)	0.73	0.17	0.46^***^		
Step 3					
Constant	0.35	1.03			
Age	-0.02	0.02	-0.09		
Gender	0.25	0.40	0.06		
Duration of concern	0.34	0.35	0.10		
Inclination to help-seeking (at baseline)	0.14	0.09	0.15		
Severity of concern (post-assessment)	0.72	0.16	0.45^***^		
Extent of exploration of the app	0.40	0.16	0.24^*^		

Additional feedback and comments received

A hundred and eight participants shared their suggestions/feedback about the app via an open-ended item on the post-assessment survey. The findings indicated that the participants engaged with the app and also reflected on what was useful/helpful. They also recommended suggestions for improving the app use experience. A brief overview of the findings is noted here. The majority of the participants provided generally positive feedback about the app. To quote some: “The app is excellent. I feel good after using it,” “It is good for students,” and “The app is useful.” Some participants also highlighted the impact of the app features, for example, “Excellent app with great content and colours” and “The design of the app is very soothing and comforting. It does help on the daily basis….”

The participants also mentioned the app's utility. The participants particularly mentioned the benefits of using the self-help sections. Some of the verbatims are “The self-help toolkits are fantastic,” “Great app to learn to apply self-help,” “The self-help sections are very helpful,” and “The guided exercises in self-help were particularly helpful in applying the concepts to my daily life.” Not only restricted to self-help, but participants also talked about how the use of the app had an impact on their overall mental health and well-being. For example, some of them said, “It is helpful to maintain a good mental health,” and “…the content is insightful and actionable, providing valuable guidance for personal growth and development. I've found myself returning to these sections regularly for motivation and clarity.” Some insightful suggestions were also brought forth by the participants. A few app navigation-related suggestions were received, for example, “I wish that the app was more easy to use, like people who have limited knowledge of smartphones might find it a bit difficult” and “App navigations can be improved.”

On the other hand, nuanced feedback about the navigation of the app was also provided, i.e., “It's not very easy to navigate through the app and once a module is complete if you refer back to the module, we have to do all the exercises again which is not very convenient. The content in the app was good though." One participant also requested that the app be made available in multiple Indian languages. Apart from the navigation-related concerns, participants also gave their feedback about the content of the app, like lessening the emphasis on writing exercises, reducing the reading material, and including more mood-regulating activities. To quote a few, “Good thought behind the app. There is too much focus on writing, I think a user will not be able to give time to that in many situations,” “Good app! For feedback, I can suggest the app to be made simpler, particularly for users like me who prefer less reading and more engaging activities,” and “Wish there were more of activities which might help in daily life, to manage the mood swings and feel more stable.”

Some participants also came up with their own suggestions to make the app more useful and interesting, for example, “Firstly, I appreciate the effort put into creating these modules as they provide valuable insights…. However, I believe there are areas where the app could be enhanced. There is room for improved visuals to aid in understanding concepts better. Adding more diagrams, infographics, or even animations could make the content more engaging and easier to grasp. When the app requires users to type, the mechanisms can be challenging to understand. Providing clearer instructions or examples for these interactive components!.” A few suggestions in terms of personalization were also voiced out: “I know it is tough to personalize an app at a larger scale but the connect between the user and app was slightly missing which can result in the user losing interest quickly” and “It would be beneficial to have an option to customize these toolkits further. For instance, I’d love to focus on stress management techniques relevant to my work environment.” One of the participants also recommended some potential topics to be added to the app (e.g., work-life balance, relationship stress, and self-care strategies).

## Discussion

Significant proportions of the study participants reported depressive and anxiety symptoms of more than one-month duration and rated their distress to be moderately severe. These observations mirror the existing research literature, which points to the high prevalence of common mental health concerns in community samples, though the study did not focus on diagnosing mental health conditions but merely characterizing the sample in terms of self-reported concerns [17-19].

High interest in learning self-help methods for managing mental health needs and reported utility of the same as reported by the participants points to the potential receptivity to structured self-help tools for mental health. The WHO pyramid model emphasizes the urgent need to offer low-intensity self-help methods to empower communities to deal with widely prevalent milder levels of mental health concerns [1]. Apps such as MindNotes are likely to address this need.

Interest in self-help methods is partly also understandable in the background of studies, which indicate a preference for self-reliance as a common barrier to seeking professional help in distressed individuals, particularly in samples of youth and young adults [20,21]. Majority of the study participants were young adults, which may be at least partly attributable to the interest of young adults in using technology-based tools apart from the sampling strategy that relied on disseminating study announcements through online mediums and outreach programs on campuses.

A bulk of the participants had not used internet-based mental health tools earlier, indicating that the participants of the present study were naïve to mental health apps. Although the use of digital technologies for varied purposes has significantly risen in India with burgeoning digital services, high smartphone penetration, and social media engagements, the use of digital health platforms for mental health is likely to be more popular for seeking online psychotherapy services following COVID-19 [22-24].

About 55% of the participants indicated that they were unsure about committing to using the app for the two-week study period while 74 (39%) participants were certain that they would do that to some extent. In line with this, slightly more than a quarter reported low rates of engagement at post-assessment in terms of extent of exploration (hardly/to a little extent) and frequency of usage of the app (hardly/once) during the two-week study period. This is understandable as the sample was a general community sample rather than specifically consisting of help-seekers, and unguided apps are known to have higher attrition/lower engagement rates than guided apps [25,26]. Although the MindNotes app has a built-in provision of connecting with a professional to clarify doubts/concerns, through a chat/voice-based professional connect section, this interaction/guidance is triggered only by the user’s action of raising a query.

About two-thirds of the participants reported using the app more than two to three times or more during the two-week period, and very few cited lack of relevance as the factor for less frequent usage. Time constraints were the most cited barrier to usage. In the background of previous literature and accounting for the fact that the MindNotes app does not involve daily practice of elements such as mood charting or mindfulness, etc., the extent of app use during this short time period seems fairly understandable, particularly in an unselected community sample [25,26].

The results suggest that the app was found to be easy to navigate and engaging by a majority of the participants, suggesting its usability. Engaging nature, ease of navigation, extent of exploration, and future likelihood of use were significantly positively correlated. High-quality content by itself is insufficient for the effectiveness of an app, unless it is sufficiently engaging for users as user-friendliness influences the level of engagement with the app content [27,28].

The present study was not an effectiveness study, but self-rated usefulness and perceived gains were examined. The app received high ratings on usefulness for self and was endorsed as relevant and useful for distressed nontreatment-seeking individuals [27]. This was further substantiated by participants endorsing gains of using the app in terms of improved awareness about mental health, self-help strategies, confidence in applying such strategies, and enhanced motivation to seek professional help. Sixty-four (59%) participants reported that they were likely to recommend the app to other individuals in their family and social circle who may be going through mental health/emotional difficulties but may have reluctance to seek professional help. All these data point to reasonably good acceptability and point to the potential utility of the MindNotes app to address the unmet needs of persons with common mental health concerns.

MindNotes from NIMHANS is a multi-module app with one of the unique modules being breaking barriers. This module focuses on identifying personally relevant barriers to seeking professional help and provides tips and strategies to overcome such barriers. Even in other modules such as self-discovery, which offers recommendations to seek help when appropriate, and in various self-help modules, attempts are made to educate and motivate the users to not only engage in self-care but also to reach out for further help and support when needed. Although the baseline survey explored the participants’ interest in various self-help modules and encouraged them to work on the same, they were free to explore the apps as they wished.

The subgroup analysis of participants with self-reported depressive and/or anxiety concerns highlighted that following engagement with the app, the inclination to seek professional help significantly increased, and this was predicted by the extent of exploration of the app over and above the severity of distress at post-assessment. This finding, though preliminary in nature, suggests the potential utility of the MindNotes app as an intervention to improve help-seeking [7].

Strengths and limitations

The MindNotes from NIMHANS app was designed by a panel of mental health professionals serving as faculty with more than three decades of clinical, community, and research experience in partnership with experts in the domain of technology with experience in the development of digital mental health platforms. The present study on this app is a preliminary one and has a few limitations. Convenient sampling was used, and the recruitment of participants was based on interest in learning self-help methods for mental well-being. There were fewer male participants, working adults, and those above 35 years of age. These factors limit the generalizability of the study findings to a diverse group of urban Indian adults and point to the need for replication studies with larger representative samples.

A time period of two weeks was given to explore the app, which may have resulted in less than optimum engagement with the app. The extent of exploration and frequency of usage indices were collected based on self-reports alone. Back-end data was not utilized to examine the usage frequency or patterns. The study, being preliminary in nature, was restricted to documenting the experience of depressive and anxiety symptoms and their duration via single self-report items. The limitations of the study notwithstanding, this preliminary study is one of the first few from India that throws light on the usability, acceptability, and perceived usefulness of a generic, indigenously developed mental health app for common mental health concerns in a community sample.

The fact that the study involved a general community sample and not a pre-selected one based on clinical symptoms or level of distress and yet observed high ratings on perceived usefulness points to the scope of the app for use by urban Indian adults interested in mental health self-help apps. Further research may also explore the potential utility of this app as a supplement to mainstream mental health services in resource-constrained clinical settings. The qualitative feedback from the participants provided leads for further enhancement of the app through improving customization, ease of navigation, and reducing need for keying in text by the users.

## Conclusions

Mental health apps can serve multiple purposes such as improving awareness and access to structured self-help resources for milder problems, improving motivation to seek professional help when needed, and serving as adjuncts to traditional mental health services. This short-term prospective study provides a preliminary demonstration of the usability, usefulness, and acceptability of an indigenous multi-module mental health app developed for Indian users with common mental health concerns. The present study findings are promising and call for large-scale, methodologically rigorous research to explore its potential to address the treatment gap in the field of mental health in low-resource settings.
